# Pharmacological LRH-1/Nr5a2 inhibition limits pro-inflammatory cytokine production in macrophages and associated experimental hepatitis

**DOI:** 10.1038/s41419-020-2348-9

**Published:** 2020-02-28

**Authors:** Juliane Schwaderer, Truong San Phan, Astrid Glöckner, Johannes Delp, Marcel Leist, Thomas Brunner, M. Eugenia Delgado

**Affiliations:** 1 Biochemical Pharmacology, Department of Biology, University of Konstanz, Konstanz, Germany; 2 In Vitro Toxicology and Biomedicine, inaugurated by the Doerenkamp-Zbinden Foundation, Department of Biology, University of Konstanz, Konstanz, Germany; 3 Cooperative Doctorate College InViTe, University of Konstanz, Konstanz, Germany

**Keywords:** Apoptosis, Mechanisms of disease, Tumour-necrosis factors, Autoimmune hepatitis, Acute inflammation

## Abstract

Liver receptor homolog-1 (LRH-1, Nr5a2) is an orphan nuclear receptor mainly expressed in tissues of endodermal origin, where its physiological role has been extensively studied. LRH-1 has been implicated in liver cell differentiation and proliferation, as well as glucose, lipid, and bile acid metabolism. In addition, increasing evidence highlights its role in immunoregulatory processes via glucocorticoid synthesis in the intestinal epithelium. Although the direct function of LRH-1 in immune cells is fairly elucidated, a role of LRH-1 in the regulation of macrophage differentiation has been recently reported. In this study, we aimed to investigate the role of LRH-1 in the regulation of pro-inflammatory cytokine production in macrophages. Our data demonstrate that pharmacological inhibition, along with LRH-1 knockdown, significantly reduced the lipopolysaccharide (LPS)-induced production of pro-inflammatory cytokines in the macrophage line RAW 264.7 cells, as well as in primary murine macrophages. This inhibitory effect was found to be independent of defects of LRH-1-regulated cell proliferation or toxic effects of the LRH-1 inhibitors. In contrast, LRH-1 inhibition reduced the mitochondrial ATP production and metabolism of macrophages through downregulation of the LRH-1 targets glucokinase and glutminase-2, and thus impairing the LPS-induced macrophage activation. Interestingly, in vivo pharmacological inhibition of LRH-1 also resulted in reduced tumor necrosis factor (TNF) production and associated decreased liver damage in a macrophage- and TNF-dependent mouse model of hepatitis. Noteworthy, despite hepatocytes expressing high levels of LRH-1, pharmacological inhibition of LRH-1 per se did not cause any obvious liver damage. Therefore, this study proposes LRH-1 as an emerging therapeutic target in the treatment of inflammatory disorders, especially where macrophages and cytokines critically decide the extent of inflammation.

## Introduction

The liver receptor homolog-1 (LRH-1/Nr5a2) belongs to the Ftz-F1 subfamily of nuclear receptors and is predominantly expressed in enterohepatic tissues of both, human, and murine organisms. Here, it is crucially involved in the transcriptional control of different physiological processes, including maintenance of stemness, proliferation, metabolism, and steroidogenesis^1^. In the liver LRH-1 controls bile acid synthesis, lipid, and glucose metabolism^2–6^, whereas in the intestinal epithelium it is involved in the control of stem cell proliferation and self-renewal through the transcriptional regulation of cyclin D1 and E1^7^. Moreover, LRH-1 controls the synthesis of immunoregulatory glucocorticoids via the expression of the steroidogenic enzymes Cyp11b1 and Cyp11a1 in the intestinal epithelium, implicating this nuclear receptor in the regulation of the local immune homeostasis^8,9^. Recently, evidence for a more direct role of LRH-1 in the control of immune cell differentiation and function has been reported. Thus far, the function of LRH-1 in immune cells has been largely ignored, mostly due to its remarkably lower expression levels when compared with endodermal tissues. While Benod et al.^10^ first reported the coincidental observation of LRH-1 expression in tumor-infiltrating immune cells in pancreatic tumor tissues, Lefevre et al.^11^ provided first evidence of the relevance of LRH-1 in cells from the hematopoietic compartment, that is, macrophages. In this study, the authors demonstrated that LRH-1 controls interleukin-13 (IL-13)-induced macrophage polarization via the Cyp1a1- and Cypb1-regulated production of the peroxisome proliferator-activated receptor-γ ligand 15-HETE (15-hydroxyeicosatetraenoic acid), highly impacting the differentiation of macrophages towards an anti-inflammatory and anti-fungal M2 phenotype^11^.

Although the role of LRH-1 in cells from hematopoietic cells is mostly unexplored, its expression appears not to be restricted to the macrophage lineage. We previously have shown that immature and mature T cells express LRH-1, which is further induced upon T cell activation^12^. A more recent publication highlights the critical role of LRH-1 in T cell maturation and functions^13^. Specific depletion of LRH-1 in T cells strongly reduced their activation-induced proliferation, resulting in the impaired induction of T cell-regulated immune responses^13^. Importantly, we identified the tumor necrosis factor (TNF) family member Fas ligand (FasL, CD95L) as an LRH-1-regulated target gene^12^. Pharmacological inhibition of LRH-1 not only resulted in reduced activation-induced *FasL* transcription and protein expression but also in attenuated FasL-mediated effector functions in T cells, such as cell-mediated cytotoxicity and activation-induced cell death. Critically, pharmacological inhibition of LRH-1 also decreased the concanavalin A-induced FasL expression in vivo, resulting in a strong protection from FasL-mediated liver cell apoptosis and associated hepatitis^12^. These studies not only demonstrate the presence and relevance of LRH-1 in the immune cell activation and effector function of hematopoietic cells but they also suggest this nuclear receptor as an interesting therapeutic target in the treatment of immunopathological diseases.

In this study, we investigated the role of LRH-1 in the regulation of lipopolysaccharide (LPS)-induced pro-inflammatory cytokines in macrophages and in an in vivo model of macrophage- and TNFα-dependent hepatitis. Our results demonstrate that inhibition of LRH-1 with two unrelated pharmacological inhibitors significantly reduced LPS-induced pro-inflammatory cytokine production in the macrophages cell line RAW 264.7, as well as in primary bone marrow-derived macrophages (BMDMs), liver-resident macrophages (Kupffer cells), and even human peripheral blood mononuclear cell (PBMC)-derived monocytes. While LRH-1 inhibition did not appear to impair macrophage survival or proliferation, we noticed a profound decrease in glutamine- and glucose-metabolizing enzymes at the transcriptional level, leading to decreased mitochondrial activity and impairment in metabolism, ultimately resulting in reduced production of pro-inflammatory cytokines. Importantly, pharmacological inhibition of LRH-1 was also able to significantly inhibit LPS-induced TNF production in vivo, thus preventing LPS/*N*-acetyl-d-galactosamine (GalN)-induced, TNF-mediated, acute hepatitis in mice. These results provide further evidence for a critical role of LRH-1 in the regulation of immune cells, in particular macrophages, and support the notion of LRH-1 as a novel and interesting pharmaceutical target in the treatment of immunopathological disorders, such as immune cell-mediated hepatitis.

## Results

### LRH-1 regulates activation-induced TNF production in splenocytes

While in the intestine LRH-1 activity has been associated with anti-inflammatory properties, for example, via the production of immunoregulatory glucocorticoids, more recent studies proposed a novel and more direct role of LRH-1 in the regulation of immune cell differentiation and effector functions^11–13^. Given that activated macrophages are a rich source of cytokines implicated in the initiation of inflammatory responses, we aimed to investigate the effect of pharmacological inhibition of LRH-1 in the activation-induced production of pro-inflammatory cytokines.

**Fig. 1:**
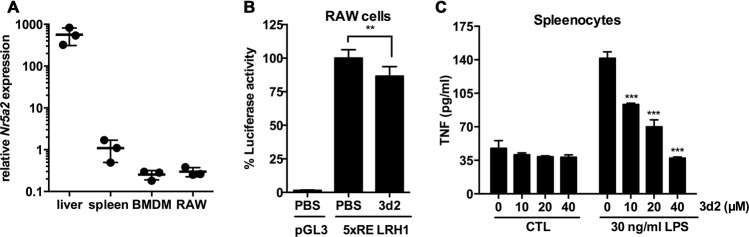
LRH-1 expression and activity in macrophages. **a** Relative *Nr5a2* mRNA expression in the liver, spleen, and bone marrow-derived macrophages (BMDM) from wild-type C57BL/6 mice, as well as in the macrophage cell line RAW 264.7 (RAW). Results are shown as relative to murine *Gapdh* mRNA expression. Mean values (bars) and individual data points from three mice, resp. cell samples, per group are shown. **b** Endogenous LRH-1-dependent transcriptional activity in RAW 264.7 cells. Cells were transfected with the LRH-1 luciferase reporter construct (5 × RE LRH-1) or an empty luciferase reporter (pGL3) and treated with vehicle (PBS) or 3d2 (40 μM). Mean values of triplicates ± SD of two independent experiments are shown. Luciferase activity was normalized to untreated cells (*t* test; **p < 0.01). **c** TNF protein levels measured in the supernatant of splenocytes from wild-type C57BL/6 mice and pre-treated for 2 h with indicated concentrations of 3d2, prior to stimulation with control buffer (CTL) or LPS (30 ng/ml). Mean values of triplicates ± SD of a representative experiment (*n* = 3) are shown (one-way ANOVA, ***p < 0.001).

We first assessed the levels of LRH-1 in macrophages and associated tissue samples. Indeed, *Nr5a2* messenger RNA (mRNA) was detected at high levels in the liver and also in spleen cells, as well as in BMDMs and in the macrophage cell line RAW 264.7, although at considerably lower expression levels (Fig. 1a). RAW 264.7 cells showed a clear endogenous LRH-1 transcriptional activity as measured by an LRH-1 activity luciferase reporter^14^, which was significantly reduced by the LRH-1 inhibitor 3d2 (Fig. 1b). 3d2 is a small inhibitor identified in a chemical screen and has been shown to directly bind to the ligand-binding domain of this nuclear receptor, stabilizing its inactive conformation and hence selectively preventing its transcriptional activity^15^. We next investigated the role of LRH-1 in activation-induced cytokine expression in macrophage containing murine splenocytes. Their stimulation with LPS resulted in the release of the pro-inflammatory cytokine TNF, which was dose dependently inhibited by 3d2 (Fig. 1c). Thus, these data confirm the fact that LRH-1 is expressed and active in macrophages, and that its pharmacological inhibition impacts LPS-induced TNF production in splenic macrophages.

### LRH-1 inhibition reduces the pro-inflammatory cytokine production in RAW 264.7 cells

**Fig. 2:**
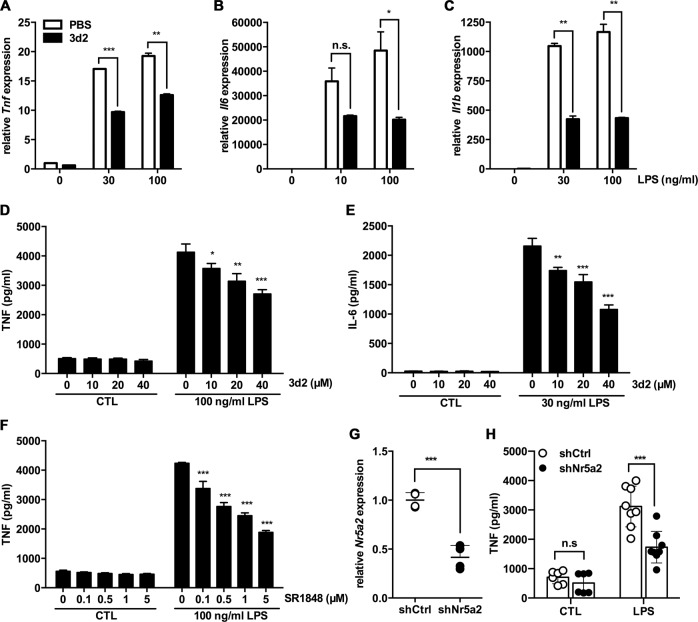
LRH-1 inhibition reduces the pro-inflammatory cytokine production in LPS-stimulated RAW 264.7 cells. mRNA expression levels of **a** *Tnf*, **b** *Il6*, and **c** *Il1b* in RAW 264.7 cells pre-treated for 2 h with vehicle (PBS) or 3d2 (40 μM) and subsequently stimulated with control buffer (CTL) or LPS at indicated concentrations for 18 h. Results are shown as relative to murine *Gapdh* mRNA expression. Mean values of triplicates ± SD of three independent experiments are shown (*t* test; **p < 0.01, ***p < 0.001). **d** TNF and **e** IL-6 secreted levels in the supernatant of RAW 264.7 cells pre-treated with indicated concentrations of 3d2 for 2 h and subsequently stimulated with control buffer (CTL) or LPS at indicated concentrations for 18 h. **f** TNF secreted levels in the supernatant of RAW 264.7 cells pre-treated for 2 h with SR1848 at indicated concentrations, prior to stimulation with control buffer (CTL) or LPS at indicated concentrations for 18 h. Mean values of triplicates ± SD of a representative experiment (*n* = 3) are shown (one-way ANOVA, *p < 0.05, **p < 0.01, ***p < 0.001). **g** Quantification of mRNA levels of *Nr5a2* in RAW 264.7 cells transduced with shRNA against Nr5a2 (shNr5a2) or shRNA control (shCtrl). Data are shown as relative to murine *Actb* mRNA and expressed as fold change to shCtrl cells. **h** TNF produced in RAW 264.7 cells transduced with shNr5a2 or shCtrl control and subsequent treated with control buffer (CTL) or LPS (30 ng/mL) for 18 h. Dots show technical replicates and bars means ± SD of data pooled from two independent experiment (two-way ANOVA, *p < 0.05, **p < 0.01, ***p < 0.001, n.s., not significant).

We next aimed to analyze the role of LRH-1 in macrophages more directly. RAW 264.7 cells stimulated with LPS strongly induced the transcription of the pro-inflammatory cytokines TNF, IL-6, and IL-1β, even at low concentrations of LPS (Fig. 2a–e). Supporting our experiments with splenocytes, a significant inhibition of LPS-induced *Tnf*, *Il6*, and *Il1b* transcripts was strongly attenuated after pharmacological inhibition of LRH-1 by 3d2 (Fig. 2a–c). In line with reduced transcription, also the LPS-induced release of TNF and IL-6 proteins were dose dependently inhibited by 3d2 (Fig. 2d, e). In order to further confirm the results obtained with 3d2, we also employed an alternative LRH-1 inhibitor, the SR1848^16^. Similarly, SR1848 also dose dependently inhibited the LPS-induced release of TNF in RAW 264.7 cells (Fig. 2f), thus further confirming the role of LRH-1 activity in macrophage-dependent cytokine production.

In addition, we employed RNA interference-mediated downregulation of LRH-1 in order to demonstrate the specificity of the LRH-1 inhibitors on LPS-induced cytokine production in macrophages. Confirming the effect of the pharmacological inhibition, LRH-1 downregulation by short hairpin RNA (shRNA) against *Nr5a2* (Fig. 2g) also resulted in a reduction of the LPS-dependent release of TNF respect untargeted controls (Fig. 2h).

### LRH-1 inhibition impairs LPS-induced cytokine production BMDMs and Kupffer cells

**Fig. 3:**
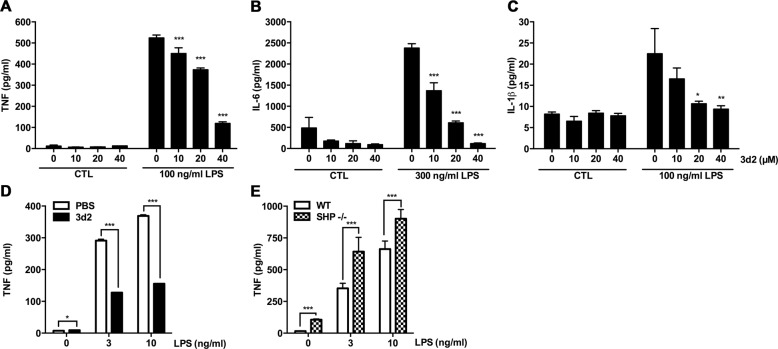
LRH-1 regulates the pro-inflammatory cytokine production in LPS-stimulated bone marrow-derived macrophages and Kupffer cells. **a** TNF, **b** IL-6, and **c** IL-1β secreted protein levels in the supernatant of bone marrow-derived macrophages (BMDMs) pre-treated for 2 h with vehicle (PBS) or 3d2 (40 μM) and subsequently stimulated with control buffer (CTL) or LPS at indicated concentrations for 18 h. Mean values of triplicates ± SD of a representative experiment (*n* = 3) are shown (one-way ANOVA *p < 0.05, **p < 0.01, ***p < 0.001). **d** TNF protein levels in the supernatant of isolated Kupffer cells from wild-type C57BL/6 mice after 2 h pre-treatment with vehicle (PBS) or 3d2 (40 μM) and subsequently stimulated for 18 h with LPS at indicated concentrations. Mean values of triplicates ± SD of one representative experiment (*n* = 3) are shown (*t* test; *p < 0.05, ***p < 0.001). **e** TNF protein levels in the supernatant of BMDMs generated from wild-type C57BL/6 (WT) or SHP-deficient (SHP−/−) mice and stimulated for 18 h with LPS at indicated concentrations. Mean values of triplicates ± SD of a representative experiment (*n* = 3) are shown (*t* test; *p < 0.05, ***p < 0.001).

As RAW 264.7 is a cell line derived from virus-transformed murine peritoneal macrophages and therefore certain pathways and associated phenotype might not be representative for untransformed cells, we extended our studies to primary macrophages. Comparable to RAW 264.7 cells, 3d2-pre-treated macrophages generated from murine BMDM showed a strong reduction of LPS-dependent TNF and IL-6 induced (Fig. 3a, b). While RAW 264.7 cells failed to release IL-1β in response to LPS (data not shown), BMDMs produced and released IL-1β protein, which was also attenuated by LRH-1 inhibition (Fig. 3c). Similarly, murine ex vivo LPS stimulation of isolated liver-resident macrophages (Kupffer cells) also resulted in increased TNF release, which was also inhibited by 3d2 (Fig. 3d). Interestingly, a similar effect was observed in primary human PBMCs. Confirming previous results in primary murine macrophages, 3d2 treatment also resulted in a decreased LPS-induced TNF production by monocytes (Supplementary Fig. 1).

In order to further support the role of LRH-1 in the regulation of macrophage activation, we generated BMDMs from small heterodimer partner (SHP, Nr0b2)-deficient mice. The nuclear receptor SHP is a transcriptional target and at the same time inhibitor of LRH-1^17,18^. In line with pharmacological inhibitors or LRH-1 downregulation, we observed increased LPS-induced TNF production in SHP-deficient macrophages compared to that in wild-type macrophages (Fig. 3e), suggesting that in the absence of SHP inhibition, a higher LRH-1 transcriptional activity results in an increased macrophage activation and TNF production.

### LRH-1 activity regulates mitochondrial metabolism in macrophages

**Fig. 4:**
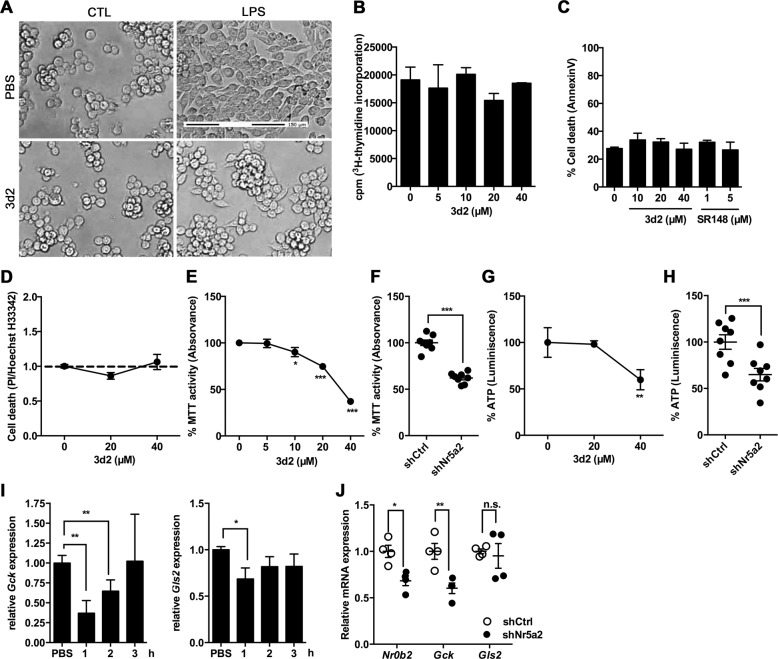
LRH-1 inhibition reduces mitochondrial activity in RAW 264.7 cells. **a** Representative microscopy images of RAW 264.7 cells pre-incubated for 2 h with 3d2 (40 μM) or vehicle (PBS), prior to stimulation with control buffer (CTL) or LPS (30 ng/ml) for 18 h (scale bar: 150 μm). **b** [^3^H]thymidine incorporation as representative of proliferation rate of RAW 264.7 cells after treatment with 3d2 at indicated concentrations. Results shown as radioactive-dependent scintillation counts per minute (c.p.m.). **c** Cell death analysis (AnnexinV staining) in 3d2- or SR1840-treated cells. **d** Cell death detection by propidium iodide (PI):Hoechst dye (H33342) ratio in 3d2-treated RAW 264.7 cells. Values have been normalized to untreated cells (dashed line). Mean values of triplicates ± SD of a representative experiment (*n* = 3) are shown. **e** MTT assay analysis of the general mitochondrial activity and **g** ATP levels in RAW 264.7 cells after overnight treatment with 3d2 at the indicated concentrations. Results are shown as normalized data to the untreated control. Mean values of triplicates ± SD of a representative experiment (*n* = 3) are shown (one-way ANOVA ***p < 0.001). **f** MTT assay analysis and **h** ATP levels in RAW 264.7 cells transduced with shRNA against *Nr5a2* (shNr5a2) or shRNA control (shCtrl), and shown as relative to untargeted controls (shCtrl). Technical replicates ± SD of a representative experiment (*n* = 3) are shown (*t* test, ***p < 0.001). **i** mRNA expression levels of *Gck* and *Gls* in RAW 264.7 cells after treatment with vehicle (PBS) or 3d2 (40 μM) at indicated time points. **j** mRNA levels of *Nr0b2* (SHP), *Gck,* and *Gls2* in RAW 264.7 cells transduced with shRNA against *Nr5a2* (shNr5a2) or shRNA control (shCtrl). Results are shown as relative to murine *βactin* mRNA expression and normalized to untreated or untargeted controls. **i** Mean values of triplicates or **j** technical replicates ± SD of two independent experiments are shown (one-way ANOVA (**i**) or *t* test (**j**), *p < 0.05, **p < 0.01, n.s. = not significant).

In line with the hypothesis of impaired LPS-mediated activation, 3d2-treated RAW 264.7 cells did not show the typical morphology of activated macrophages upon LPS stimulation. This morphology consist of extensive cell spreading or lamellipodia on the leading edge of the cells^19^ (Fig. 4a). This suggested that LRH-1 inhibition results in reduced macrophage activation, ultimately resulting in reduced cytokine production.

[^3^H]thymidine incorporation assays showed no obvious inhibition of proliferation in RAW 264.7 cells treated with different concentrations of 3d2 (Fig. 4b), disregarding the hypothesis that the 3d2-dependent effect in cytokine production might be due to a reduced cell proliferation after pharmacological LRH-1 inhibition, as observed previously^13^. Moreover, neither 3d2 nor SR1848 inhibitor had direct cytotoxic effects in RAW 264.7 cells, as analyzed by AnnexinV staining and flow cytometry (Fig. 4c), or by the propidium iodide (PI)/Hoechst dye-stained ratio (H33342)^20^ (Fig. 4d). Interestingly though, when cell viability was monitored with the MTT (3-(4,5-dimethylthiazol-2-yl)-2,5-diphenyltetrazolium bromide assay, RAW 264.7 cells treated with 3d2 showed a dose-dependent decrease in general mitochondrial activity as documented by the reduced production of the MTT metabolite, the insoluble formazan salt (Fig. 4e). Indeed, besides regulating cell cycle progression and proliferation, LRH-1 has been involved in the control of mitochondrial function and metabolic processes^1–3,21,22^. In line with this notion, RAW 264.7 cells exposed to increasing concentrations of 3d2 showed a significant decrease in cellular ATP levels (Fig. 4g). Thus, LRH-1 activity may also regulate mitochondrial metabolism in macrophages. Interestingly, this hypothesis could be confirmed by the shRNA-mediated downregulation of LRH-1 (Fig. 2g). In this case, shRNA against *Nr5a2* resulted also in a decrease in MTT activity (Fig. 4f) and in ATP levels (Fig. 4h), when compared to untargeted controls.

In the liver, LRH-1 has been described as a critical transcriptional regulator of the mitochondrial glutaminase-2 enzyme (Gls2), greatly impacting on the glutamine-induced metabolic signaling pathway in hepatic cancer cells^2^. Similarly, LRH-1-dependent regulation of glucokinase (Gck) has been described to tightly control liver glucose sensing and processing, since Gck is the enzyme responsible for catalyzing the first step of glycolysis^3^. In line with the role of LRH-1 in regulating these gene products, we observed reduced levels of *Gck* and *Gls2* transcripts in RAW 264.7 cells exposed to 3d2 (Fig. 4i). Similarly, downregulation of LRH-1 resulted in reduced levels of the LRH-1 targets *Nr0b2* (SHP), *Gck*, and to lesser extent *Gls2* (Fig. 4j). These results suggest that reduced LRH-1-dependent glucose and glutamine metabolism could contribute to the inhibition of cytokine production in LPS-stimulated macrophages.

### LRH-1 inhibition impairs the LPS-dependent increase in glycolysis and glutaminolysis resulting in reduced macrophage cytokine production

**Fig. 5:**
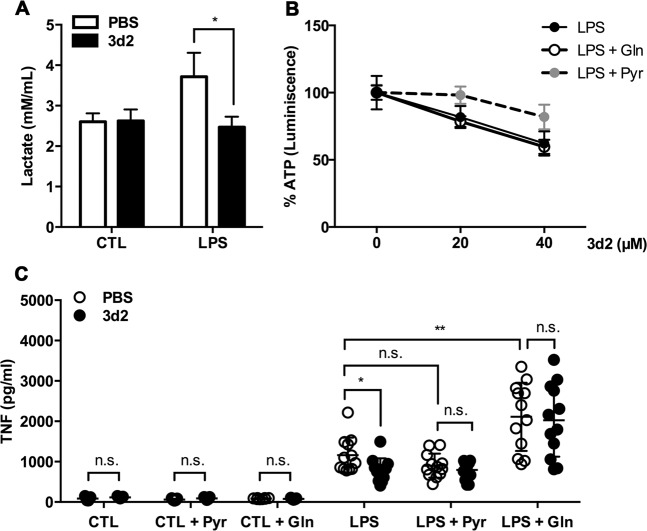
LRH-1 inhibition limits glycolysis and glutaminolysis in RAW 264.7 cells. **a** Lactate produced in RAW 264.7 cells supernatant after 2 h pre-treatment with 3d2 (40 μM) or control buffer (PBS), and subsequently stimulated with control buffer (CTL) or LPS (30 ng/ml) for 18 h. Mean values of triplicates ± SD of a representative experiment (*n* = 2) are shown (*t* test; *p < 0.05). **b** ATP levels of RAW 264.7 cells after pre-treatment for 2 h with control buffer (CTL) or 3d2 at indicated concentrations in the medium at normal conditions, or medium supplemented with 8 mM pyruvate (Pyr) or 4 mM l-glutamine (Gln), following 18 h stimulation with LPS (30 ng/ml). The percentage of ATP was normalized to the untreated control. Mean values of triplicates ± SD of a representative experiment (*n* = 3) are shown. **c** TNF levels in the supernatant of RAW 264.7 cells pre-treated for 2 h with vehicle (PBS) or 3d2 (40 μM), following 18 h stimulation with control buffer (CTL) or LPS (30 ng/ml) in normal culture medium, or medium supplemented with 8 mM pyruvate (Pyr) or 4 mM Gln. Pooled values (three to four technical replicates) of three independent experiments ± SD are shown (*t* test or two-way ANOVA; *p < 0.05, **p < 0.01, n.s. = not significant).

Regulation of glutamine, glucose, and fatty acids metabolic pathways is essential for energy production and macromolecule biosynthesis, also in macrophages^23^. Interestingly, depending on environmental conditions and stimuli, macrophages are able to reprogram their metabolic pathways in order to cope with different requirements and functions, for example, pro- or anti-inflammatory activities. For example, hallmarks of LPS-activated macrophages are enhanced glycolysis, increased pentose-phosphate pathway, and fatty acid synthesis, as well as impaired mitochondrial oxidative phosphorylation and reduced TCA (tricarboxylic acid) cycle with the accumulation of citrate and succinate^23^. This effect, known as aerobic glycolysis or the Warburg effect^24^, allows these macrophages to support their demand for energy and specific metabolites, in order to sustain their high secretory and phagocytic function^25^. Accordingly, LPS stimulation of RAW 264.7 cells resulted into increased levels of lactate production, indicating enhanced glycolysis. Interestingly, this effect was reduced when LRH-1 was inhibited by 3d2 (Fig. 5a). Thus, it seems feasible that the 3d2-mediated downregulation of the LRH-1 targets *Gck* and *Gls2* might contribute to the reduced glycolysis and glutaminolysis in LPS-activated macrophages, resulting in impaired macrophage activation and cytokine production.

In order to confirm this hypothesis, we investigated whether supplementation of the culture medium with either pyruvate (Pyr) or l-glutamine (Gln) could restore cellular ATP levels and LPS-induced cytokine production. Remarkably, only Pyr, but not Gln, supplementation was able to restore the reduced ATP levels upon 3d2 treatment (Fig. 5b). However, either Gln or Pyr supplementation could reverse the 3d2-mediated inhibition of LPS-induced TNF production in RAW 264.7 cells (Fig. 5c). These results indicate that pharmacological inhibition of LRH-1 indeed impacts cellular glycolysis and glutaminolysis in macrophages, which impairs LPS-induced cytokine production. However, as Gln supplementation was not only able to neutralize the 3d2-mediated inhibition on LPS-induced TNF production, but to even further increase it, Gln-dependent anaplerosis seems to be more critical in the regulation of macrophage effector functions than intracellular Pyr or ATP levels.

Stimulation of TLR4 by LPS results in the rapid activation of mitogen-activated protein (MAP) kinase and nuclear factor-κB (NFκB), and the transcriptional regulation of the pro-inflammatory TNF, IL-6, and IL-1β^26^. Also, LPS stimulation of BMDMs promoted the phosphorylation and activation of the MAP kinases ERK1/2 (extracellular signal-regulated kinase 1/2) and p38, as well as the activation of NF-κB, as illustrated by the degradation of the NF-κB inhibitor, IκBα (Supplementary Fig. 2A). In line with the relevance of these pathways, a significant reduction of TNF production was observed in LPS-stimulated macrophages upon treatment with the MAP kinase inhibitor U0126, along with a strong inhibition of ERK activation and a partial inhibition of p38 (Supplementary Fig. 2A, B). Similarly, also macrophage pre-treatment with 3d2 resulted in reduced MAP kinase activation and IκBα degradation (Supplementary Fig. 2A), although to a lower degree than U016, which correlated well with the inhibition of LPS-induced TNF production by these two inhibitors (Supplementary Fig. 2B).

### 3d2-mediated LRH-1 inhibition protects from TNF-dependent hepatitis

**Fig. 6:**
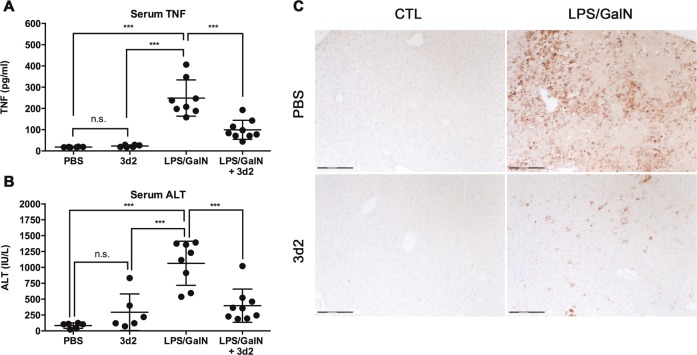
LRH-1 inhibition protects from TNF-dependent hepatitis. Wild-type C57BL/6 mice (*n* = 6–9 per group) were pre-treated with vehicle (PBS) or 3d2 (50 mg/kg body weight) i.p. injections for 1 h and then challenged by i.p. injections of LPS (0.2 mg) and GalN (40 mg). Serum was harvested for the analysis of **a** TNF protein concentrations and **b** transaminase (ALT) levels (*t* test; ***p < 0.001, n.s. = not significant). **c** Immunohistochemical detection of cleaved caspase-3-positive, apoptotic hepatocytes in liver sections from untreated (CTL) or LPS/GalN i.p. injected mice pre-treated with PBS or 3d2 as described above (scale bar: 150 μm).

Given the important role of TNF in the pathogenesis of various immunopathological disorders^27^, we next aimed to investigate whether inhibition of LRH-1 could be used to pharmacologically target macrophage-mediated, TNF-dependent tissue destruction. Simultaneous intraperitoneal (i.p.) injection of LPS and the hepatic transcriptional inhibitor GalN in wild-type C57BL/6 mice leads to massive LPS-induced TNF production by liver LPS-activated macrophages and GalN-mediated sensitization of hepatocyte to TNF-induced apoptosis, resulting in massive liver damage and associated hepatitis^28^. Indeed, LPS/GalN injection caused a remarkable increase in serum TNF (Fig. 6a), serum transaminase (alanine aminotransferase (ALT)) (Fig. 6b), and hepatocyte apoptosis (Fig. 6c). Excitingly, treatment of mice with 3d2 not only reduced LPS-induced TNF levels but also significantly lowered liver damage as documented by lower serum ALT and reduced cleaved caspase-3-positive hepatocytes (Fig. 6a–c). Of interest, even though hepatocytes express high levels of LRH-1 (Fig. 1a), treatment of mice with 3d2 alone caused only a moderate increase in serum transaminases (Fig. 6b) and no hepatocyte apoptosis could be observed. Our results indicate that pharmacological inhibition of LRH-1 using indicated 3d2 concentrations is relatively well tolerated by the liver, and opens a window of opportunity to target LRH-1 pharmacologically during inflammatory disorders with a major macrophage contribution.

In summary, we here show that LRH-1 is expressed in macrophages, and critically contributes to LPS-induced expression and secretion of pro-inflammatory cytokines. We provide further evidence that pharmacological targeting of LRH-1 results in reduced TNF-dependent hepatitis. Finally, this study supports the notion that LRH-1 has important, so far underestimated function in the regulation of the immune system^12,13^.

## Discussion

The important role of LRH-1 in physiological as well as pathophysiological processes in endodermal tissues is well established, and thus the development of drugs able to manipulate LRH-1 activities is under extensive research. For example, the role of LRH-1 in the regulation of lipid and glucose metabolism in the liver is subject to experimental targeting during metabolic syndrome and in type 1 and 2 diabetes^2–6,21,29^. Similarly, LRH-1 is overexpressed in many tumors of endodermal tissues, such as hepatocellular carcinoma, pancreatic tumors, and colorectal cancers, where it drives proliferation via the transcriptional control of cell cycle-regulating genes, such as cyclins D1 and E1^7,10,30–32^. Thus, LRH-1 inhibitors may have great potential in the treatment of these tumors^33^. In this regards, 3d2 and SR1848 have been shown to represent interesting lead compounds, which rather selectively inhibit LRH-1, although by different mechanisms and with different potencies^12,15,16^.

As reported by our group and others, increasing evidence revealed that LRH-1 is not only present in endodermal tissues and tumors but it is also expressed at lower levels in immune cells (see Fig. 1 and refs. ^10–13^). Substantially lower expression levels do, however, not necessarily mean that LRH-1 is less relevant in immune cells. Of interest, although LRH-1 is strongly expressed in the liver and intestine, where it fulfills important functions, deletion of LRH-1 in these tissues still results in functional liver, resp. intestine, even though certain processes are diminished or altered^5,8,21,34^. In marked contrast, we recently demonstrated that the deletion of LRH-1 in T cells, although only expressed at very low levels, results in a drastic reduction in absolute T cell numbers, and particularly, in the inability to mount protective immune responses or immunopathologies^13^. This stronger LRH-1 dependency of immune cells compared to endodermal tissue offers an interesting window of opportunity. Thus, while systemic deletion of LRH-1 is lethal^35^, the transient use of LRH-1 inhibitors could be helpful to treat acute episodes of inflammation and immunopathologies, yet leaving LRH-1-positive tissue cells mostly unaffected. It is interesting to point out that acute treatment of mice with the LRH-1 inhibitor 3d2 did not cause any significant liver damage, yet was able to reduce macrophage-induced and TNF-mediated acute hepatitis (Fig. 6). Similarly, we have previously shown that 3d2 can inhibit FasL expression in T cells and thereby reduce T cell-mediated liver damage and hepatitis^12^. These findings illustrate the great potential of LRH-1 inhibitors in the treatment of immune cell-mediated pathologies.

In this study, we not only show that LRH-1 has important function in macrophages in the regulation of LPS-induced cytokine expression, but also we show that LRH-1 is druggable in vitro and in vivo, leading to reduced expression and release of pro-inflammatory cytokines and associated immunopathologies. It partly confirms in part a recent publication by Lefrevre and colleagues^11^ demonstrating an important role for LRH-1 in IL-13-induced macrophage polarization and effector functions. Even though this report rather suggested an anti-inflammatory role of LRH-1 in M2 macrophages, our study on LPS-induced expression of pro-inflammatory cytokines points out that LRH-1 may also have an important role in the activation of M1 macrophages. It therefore seems that the role of LRH-1 in the regulation of differentiation, polarization, and macrophage effector functions is much broader than initially thought. Clearly, more detailed studies will be required to understand the relative role of LRH-1 in various aspects of macrophage biology.

How does LRH-1 inhibition affect the LPS-induced expression of pro-inflammatory cytokines? A reasonable first guess would be that as a transcription factor LRH-1 directly regulates the expression of TNF, IL-6, and IL-1β. We have previously shown that FasL, a close homolog of TNF, is a direct transcriptional target of LRH-1^12^. Yet so far, we failed to identify obvious LRH-1-binding sites in the *Tnf*, *Il6*, and *Il1b* promoters. Thus, LRH-1 appears to regulate macrophage functions in a more general manner and upstream of the transcription of these cytokines. An interesting hint comes from the observation that pharmacological inhibition of LRH-1 prevents the LPS-induced morphology of activated macrophages (Fig. 4). Similarly, we have seen that the general mitochondrial activity (MTT), as well as the ATP concentration, is drastically reduced in 3d2-treated cells. Indeed, loss of LRH-1 has been very recently related to reduced mitochondrial function and ATP production in hepatocytes^22^. Thus, it is very likely that LRH-1 inhibition results in reduced or altered metabolism in LPS-stimulated macrophages, which impairs downstream effector functions, such as the secretion of pro-inflammatory cytokines. In line with this notion, we have seen that the expression of established LRH-1 target genes *Gck* and *Gls2* (Fig. 4) were reduced upon treatment with 3d2. Furthermore, culture medium supplementation with Pyr or Gln was able to restore 3d2-mediated inhibition of LPS-induced TNF production (Fig. 5). As the addition of Gln had a general enhancing effect on LPS-induced TNF expression, it is likely that LRH-1-regulated mitochondrial anaplerosis is more critical for these processes than cellular ATP levels, as being restored by Pyr (Fig. 5). In this regard, when glycolytic rates are reduced, as observed in 3d2-treated macrophages, alternative metabolites can fuel the TCA cycle in order to cope with the LPS-induced metabolic requirements^36^.

Although changes in energy metabolism have a great impact on vital functions of cells, such as proliferation and death/survival, we were able to exclude impaired proliferation or cytotoxicity as one of the underlying reasons for reduced expression of pro-inflammatory cytokines in 3d2-treated macrophages (Fig. 4). Thus, it is likely that other pathways or processes must be involved. Given the importance of MAP kinase pathways and NF-κB activation in the LPS-induced expression of pro-inflammatory cytokines^26^, and the reduced activation of these signaling processes in 3d2-treated macrophages (Supplementary Fig. 2), it is very likely that LRH-1-regulated metabolic processes, including but not restricted to *Gls2* and *Gck* regulation, are involved in the fine-tuning of these signaling cascades. Yet, how these processes are linked is incompletely understood. LPS-induced macrophage polarization has been shown to highly relay on Pyr dehydrogenase activity, suggesting a higher demand for glucose-dependent Pyr production. In this regard, Meiser et al.^23^ were able to inhibit LPS-dependent TNF production by using a specific Pyr transporter inhibitor (UK5099). Similarly, in LPS-stimulated macrophages Gln is consumed at high rate and enters the TCA cycle through glutaminolysis, mostly contributing to succinate accumulation^37^. Interestingly, inhibitors affecting succinate levels in LPS-stimulated macrophages greatly impact the stabilization and activation of the hypoxia-inducible factor-1α^25,38^, and thereby the induction of its transcriptional target IL-1β, while TNF remains unaffected^37^. This result suggests specific metabolite requirements for the proper cytokine production in LPS-stimulated macrophages. Of note, glycolysis inhibition with 2-deoxyglucose, a glucose derivative unable to undergo glycolysis, also results in reduced succinate levels and LPS-induced IL-1β expression, suggesting that both glycolysis and glutaminolysis contribute to the regulation of effector functions in LPS-stimulated macrophages^37,39^. As these effects are, however, restricted to LPS-induced IL-1β expression, they cannot explain the effect of LRH-1 inhibition on TNF and IL-6 expression. Thus, alternative LRH-1-regulated pathways must exist in macrophages and remain to be identified.

In summary, our study demonstrates a novel role for LRH-1 in the regulation of LPS-induced cytokine expression in macrophages. Furthermore, the successful inhibition of TNF-mediated hepatitis proposes LRH-1 inhibition as a novel therapeutic approach in the treatment of acute inflammatory disorder.

## Materials and methods

### Reagents

The LRH-1 inhibitor 3d2 was re-synthesized according to the publication of Benod et al.^15^ at ChemBridge Corp (San Diego, CA, USA). The LRH-1 inhibitor SR1848 was obtained from Sigma-Aldrich (Steinheim, Germany) and the MAPK kinase (MEK) inhibitor U0126 from New England Biolab (Ipswich, MA, USA). Gln and LPS was from *Salmonella minesota* strains and GalN were purchased from Sigma-Aldrich (Steinheim, Germany).

### Mice

Male and females wild-type C57BL/6 mice and SHP−/− mice were used between 10 and 12 weeks for ex vivo and in vivo experiments. Mice were bred and kept in the animal facility of the University of Konstanz. All animal experiments complied with animal experimentation regulations of Germany and were approved by the Ethics Review Committee of the regional council.

### Cell culture

RAW 264.7 cells, a SV40 virus-transformed peritoneal macrophage cell line from a male BALB/c mouse, was obtained from ATCC and maintained in Dulbecco’s modified Eagle’s mMedium (DMEM) medium (Sigma). All cell culture media were supplemented with 10% fetal calf serum (Sigma) and 50 μg/ml gentamicin (Sigma), and in some experiments, the medium was supplemented with 4 mM Gln or 8 mM Pyr.

### Culture and differentiation of human PBMC-derived monocytes

Blood from healthy donors was collected on the same day as the assays were performed. The blood was diluted 1:1 with phosphate-buffered saline (PBS). Afterwards, diluted blood was layered on the top of a Ficoll-Hypaque solution (HISTOPAQUE^®^-1077, Sigma) and PBMCs were isolated after density gradient centrifugation. The cells were washed twice with Hank’s balanced salt solution (Sigma) 2 × 10^6^ cells were seeded into 75-cm^2^ flasks. Monocyte enrichment was performed by adherence for 1 h at 37 °C in a humidified incubator 5% CO_2_. Non-adherent cells were removed by washing the flasks twice with RPMI.

### Culture and differentiation of BMDMs

C57BL/6 wild-type and SHP−/− mice were sacrificed for the isolation of marrow cells from femur and tibia bones. The bones were cut at both ends open and flushed with DMEM medium. Bone marrow cells were passed through a cell strainer and washed with DMEM medium in order to generate single-cell suspension. Cell numbers were adjusted to 2 × 10^6^ cells/ml and plated in 10-cm culture dishes for macrophage differentiation. Bone marrow macrophage progenitors were differentiated in complete DMEM medium containing 10% L929-conditioned medium for 7 days. In between, cells were washed twice with PBS every 2 days and fresh medium was added afterwards. After 7 days, fully differentiated macrophages were washed with PBS and non-macrophages were removed by incubation with 0.25% trypsin/EDTA for 5 min. Macrophages were harvested by incubation with fresh 0.25% trypsin/EDTA for 20 min and seeded for further experiments. Fully differentiated cells were seeded into 96-well plates (60,000 cells/well). After overnight adherence, cells were treated with respective inhibitors and stimulated with LPS.

### Isolation of Kupffer cells

Kupffer cells were enzymatically dissociated from murine liver samples using a two-step enzymatic microperfusion technique with collagenase, as described previously^40^. Kupffer cells were cultured in DMEM containing 10% FCS, 4mM Gln, and 30 μg/ml gentamicin at 37 °C and 5% CO_2_.

### Transfection/transduction

RAW 264.7 cells were passaged 24 h prior to transfection at a ratio of 1:3. A total of 2.0 × 10^6^ cells were transfected with 6 µg of plasmid DNA in a Gene Pulser Cuvette (Bio-Rad) by electroporation using an Amaxa Nucleofector Device (Lonza). Electroporated cells were pooled in complete medium and seeded into a tissue culture dish for further experiments.

LRH-1 downregulation in RAW 264.7 cells was performed using lentiviral expression vectors containing shRNA sequence against murine LRH-1 (shNr5a2, clone ID: NM_030676.1-1160s1c1, Sigma-Aldrich) or corresponding untargeted control. Cells were selected with puromycin (Sigma-Aldrich) for at least 2 days prior to experiments.

### Reporter assays

RAW 264.7 cells were transiently transfected with expression and luciferase reporter plasmids. The LRH-1 reporter containing five copies of the LRH-1 response element has been described previously^14^ (Promega). Co-transfection of β-galactosidase expression plasmid (Invitrogen) served for normalization. One day after transfection, cells were either control treated or treated with different concentrations of the LRH-1 inhibitors for 18 h. Cells were then lysed, and luciferase and β-galactosidase activity was measured in cell lysates as described previously^41^.

### RNA isolation and quantitative PCR

For RNA isolation, cells were lysed in 1 ml peqGOLD TriFast reagent (PeqLab) and RNA was isolated according to the manufacturer’s protocol. One microgram of RNA was reverse transcribed using a High-Capacity cDNA Reverse Transcription Kit (Applied Biosystems) and cDNAs (complementary DNAs) were used for quantification of gene expression by quantitative real-time PCR using FAST SYBR Green Master Kit System. Murine *Nr5a2* and corresponding *Actb* transcripts were detected using a FAM-based TaqMan probe and the Volcano3G RT-PCR Probe Master Mix (myPols Biotec GmbH). All of them were quantified by using a StepOnePlus Real-time PCR System (Applied Biosystems). All gene-specific primers were designed to span an exon–exon junction to detect specific mRNA transcripts. Gene expression was normalized using murine *βActin* or murine *Gapdh*. The following primers were used: murine *βActin*: 5′-TATTGGCAACGAGCGGTTCC-3′ (forward), 5′-GCACTGTGTTGGCATAGAGG-3′ (reverse); murine *Gapdh*: 5′-CGTCCCGTAGACAAAATGGT-3′ (forward), 5′-TCTCCATGGTGGTGAAGACA-3′ (reverse); murine *Il1b*: 5′-TGCCACCTTTTGACAGTGATG-3′ (forward), 5′-ATGTGCTGCTGCGAGATTTG-3′ (reverse); murine *Il6*: 5′-CACAAGTCCGGAGAGGAGAC-3′ (forward), 5′-TTGCCATTGCACAACTCTTT-3′ (reverse); murine *Tnf*: 5′-TAGCCCACGTCGTAGCAAAC-3′ (forward), 5′-ACAAGGTACAACCCATCGGC-3′ (reverse); murine *Nr0b2* from Quantitec primers, GeneGlobe, Qiagen (NM_011850, # 249900*)*; murine *Gck*: ACATTGTGCGCCGTGCCTGTGAA (forward), AGCCTGCGCACACTGGCGTGAAA (reverse); murine *Gls2*: GACCGTGGTGAACCTGCTAT (forward), TGCGGGAATCATAGTCCTTC (reverse). murine *Nr5a2*: CGCATGGGAAGGAAGGGACAATCT (probe), GCTGGAGTGAGCTCTTGATT (forward), GTGTGAGATGATGGTGGAGTAG (reverse); murine *Actinb*: probe TGGCATTGTTACCAACTGGGACGA (probe), GAGGTATCCTGACCCTGAAGTA (forward), CACACGCAGCTCATTGTAGA (reverse).

### Enzyme-linked immunosorbent assay (ELISA)

Cells were pre-treated with LRH-1 inhibitors for 2 h and then were stimulated with LPS for 18 h. Plates were centrifuged and supernatant was collected. The following cytokines were detected by using ELISA-based matched antibody pairs (BioLegend); murine TNF (clone TN3-19.12, # 506102, 3 μg/ml, BioLegend and clone Poly5160, # 516003, 1 μg/ml, BioLegend) or human TNF (clone MAb1, # 502802, 1 μg/ml, BioLegend and clone Mab11, BioLegend # 502904, 0.5 μg/ml), IL-6 (clone MP5-20F3, # 504502, 1 μg/ml, BioLegend and clone MP5-32C1, # 504602, 1 μg/ml, BioLegend). IL-1β was analyzed with the DuoSet ELISA Development Kit Mouse IL-1β (R&D Systems, # DY401-05) according to the manufacturer’s instructions. For the detection of the secondary antibodies, streptavidin conjugated to horseradish peroxidase (Calbiochem, # 189733) and TMB substrate (BioLegend # 421101) were used following the manufacturer’s instruction. The reaction was stopped by the addition of 1 M H_2_SO_4_ and cytokine concentration was quantified by measuring the absorbance at a wavelength of 450 nm (reference wavelength 570 nm) with a microplate absorbance reader (Sunrise Tecan Reader).

### MTT respiration assay and ATP measurement

MTT (Sigma-Aldrich) solution was added to the RAW 264.7 cell culture to a final concentration of 500 μg/ml and incubated for 1–2 h (37 °C, 5% CO_2_). After discarding the medium, MTT was solubilized in 100 µl of dimethyl sulfoxide (DMSO) (Roth) per well (15 min, room temperature) and the absorbance was measured on a microplate absorbance reader (Sunrise Tecan Reader) at 562 nm. The decrease in cell respiration (%) was calculated as 100 × (1 − (OD exp. mean value (− substrate blank)/OD control mean value (− substrate blank)). Cellular ATP measurements were performed using the CellTiter-Glo 2.0 Kit (Promega) according to the manufacturer’s instructions.

### Lactate measurement

Lactate concentrations in RAW 264.7 cell supernatants were determined by using LAC 142 lactate test (Diaglobal GmbH), according to the manufacturer’s instructions. LPS-treated controls were used as a reference.

### Western blot analysis

BMDMs were treated for 2 h with control buffer (UT), 3d2 (40 μM), or the MEK inhibitor U0126 (10 μM; Sigma-Aldrich) and then stimulated with 100 ng/ml LPS for 60 min. Cells were then lysed in NP-40 lysis buffer (150 mM NaCl, 50 mM Tris, pH 7.6, 1 mM EDTA, and 1% NP-40) and lysates were separated on a denaturing 12% sodium dodecyl sulfate-polyacrylamide gel electrophoresis gel. After transfer to polyvinylidene difluoride membranes (Roche), membranes were incubated overnight at 4 °C with antibodies against phospho-specific ERK1/2 (# 9101), phospho-p38 (# 4511), c-Jun N-terminal kinase (# 9252), and IκBα (# 9242) antibodies (1:1000; Cell Signaling Technology) or mouse anti-tubulin (# T5168, 1:4000; Sigma-Aldrich) as a loading control.

### Detection of cell death by AnnexinV staining

Cells were exposed to 3d2 (0–40 μM) or SR148 (0–5 μM) overnight, harvested, and resuspended in AnnexinV binding buffer (10 mM HEPES, 150 mM NaCl, 5 mM KCl, 1 mM MgCl_2_, 1.8 mM CaCl_2_) containing 1 µg/ml fluorescein isothiocyanate-labeled AnnexinV. Cell suspensions were analyzed on a LS Fortessa cytometer (BD Biosciences). A total of 10,000 cells were analyzed for each sample. The percentage of AnnexinV-positive apoptotic cells with respect to total cells was determined using the FlowJo software (version 10).

### Detection of cell death by PI/Hoechst dye staining

Cells cultured in phenol-red free medium were exposed to 3d2 (0–40 μM) overnight, and then stained with PI and Hoechst H33342 at a final concentration of 1 μg/ml each. Staining solution (dyes in PBS) was directly added to the culture medium. Cells were stained for 30 min at 37 °C, 5% CO_2_ for subsequent analysis. Top-well measurements were performed in a microplate fluorescence reader (Sunrise Tecan Reader), where excitation and emission wavelengths for PI were 535 and 617 nm and for H33342, 361 and 486 nm, respectively, as described previously^20^.

### [^3^H]thymidine incorporation assay

At 24 h prior to the treatment, 2 × 10^4^ cells per well were seeded into a 96-well plate. After 6 h with 3d2 treatment, cells were labeled with 0.5 µCi [methyl-^3^H]thymidine (Hartmann Analytic) per well for 18 h in a humidified incubator with 5% CO_2_ at 37 °C. Afterwards, ^3^H-thymidine-labeled DNA was harvested onto glass fiber filters using Omnifilter-96 cell harvester (PerkinElmer). The amount of radioactivity incorporated into DNA and proportional to the amount of proliferating cells was measured using a Top Count Microplate Scintillation Counter (PerkinElmer). The readout was expressed as counts per minute (c.p.m.) per well.

### LPS/GalN-induced hepatitis

LPS and GalN were diluted in sterile endotoxin-free PBS. Wild-type C57BL/6 male mice were pre-treated with 3d2 (50 mg/kg body weight, dissolved in 10% DMSO in PBS) by i.p. injection 1 h before the LPS/GalN i.p. injection (100 ng/ml, 20 mg/ml in 200 μl). Six hours after the challenge, mice were sacrificed and serum samples were collected. Liver samples were fixed in formalin, dehydrated, and paraffin embedded for histological analysis or cleaved caspase-3 immunohistochemistry.

### Transaminase assay

For quantitative determination of ALT activity in the serum of mice, an ALT Reagent Colorimetric Endpoint Method Kit (Teco Diagnostics) was used according to the manufacturer’s recommendation with 1:5 diluted sera.

### Histology and immunohistochemistry

Formalin-fixed and paraffin-embedded liver tissues sections were counter-stained with hematoxylin and eosin staining for histological analysis. Apoptotic cells in tissue sections were detected using an anti-cleaved caspase-3 antibody (Clone Asp175, Cell Signaling Technology), as described previously^42^. All sections were observed and photographed with the PALM MicroBeam microscope (Zeiss).

### Statistical analysis

Student’s unpaired *t* test and ordinary one-way analysis of variance (ANOVA) or two-way ANOVA were performed using the Prism7 software (GraphPad Software) to identify significant differences between experimental groups. Bonferroni was the multiple comparison test used when needed. A *p* value of >0.05, <0.05, <0.01, and < 0.001 was regarded significant and marked as n.s., *, **, and ***, respectively.

## Supplementary information

Supplementary Figure 1 & Figure 2
