# Supplementary material for: Pharmacological LRH-1/Nr5a2 inhibition limits pro-inflammatory cytokine production in macrophages and associated experimental hepatitis
Source: Cell Death Dis. 2020 Feb 28;11(2):154. doi: 10.1038/s41419-020-2348-9 (PMC7048823; doi:10.1038/s41419-020-2348-9)
Supplement: Supplementary file 1 — Supplementary Figure 1 & Figure 2 [file 41419_2020_2348_MOESM1_ESM.docx]

**Supplementary Material**


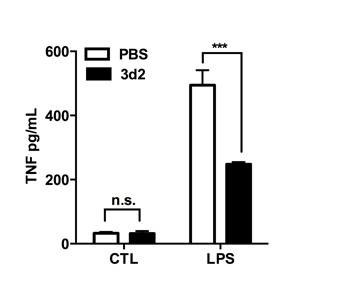


**Supplementary Figure 1: Effect of 3d2 in the LPS-activated human PBMC-derived monocytes.**

Quantification of TNF produced in human PBMC-derived monocytes pre-treated with vehicle (PBS) or 3d2 (40 μM) for 2 h and subsequent treated with control buffer (CTL) or treated with LPS (100 ng/ml) overnight. Data shows means ± SD of one experiment (n = 2) (Two-way ANOVA, *p < 0.05, **p < 0.01, ***p < 0.001, n.s. not significant).


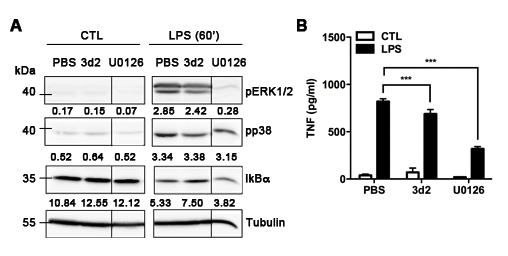


**Supplementary Figure 2: Impaired LPS-induced activation of signaling pathways in 3d2-treated BMDM.**

(**A**) Phospho-ERK1/2 (pERK1/2), phoshpo-p38 (pp38) and IκBα protein levels in BMDM 2h pre-treated with vehicle (PBS), 3d2 (40 μM) or the MEK inhibitor U0126 (10 μM) for 2 h and subsequently stimulated with control buffer (CTL) or 100 ng/ml LPS for 60 min. Tubulin was used as loading control. Values below images show densitometry analysis quantification (**B**) TNF levels in the supernatant of BMDM pre-treated with vehicle (PBS), 3d2 (40 μM) or the MEK inhibitor U0126 (10 μM) for 2 h and stimulated with control buffer (CTL) or LPS (100 ng/ml) for 18 h. Mean values of triplicates ± SD of a representative experiment (n=2) are shown (one-way ANOVA ***p < 0.001).
